# Hatching asynchrony *vs*. foraging efficiency: the response to food availability in specialist *vs*. generalist tit species

**DOI:** 10.1038/srep37750

**Published:** 2016-11-28

**Authors:** R. Barrientos, J. Bueno-Enciso, J. J. Sanz

**Affiliations:** 1 Área de Zoología, Departamento de Ciencias Ambientales, Facultad de Ciencias del Medio Ambiente, Universidad de Castilla-La Mancha, Avenida Carlos III, s/n, E-45071, Toledo, Spain; 2 Departamento de Ecología Evolutiva, Museo Nacional de Ciencias Naturales (MNCN-CSIC), José Gutiérrez Abascal, 2, E-28006, Madrid, Spain

## Abstract

Breeding mistiming is increasingly frequent in several ecosystems in the face of current climate change. Species belonging to higher trophic levels must employ mechanisms to reduce it. One of these mechanisms is hatching asynchrony, with the eggs in a clutch hatching over a period of several days. Some authors have suggested it to be adaptive when food is unpredictable. However, these birds can also suffer associated costs. We tested whether a species with higher foraging efficiency avoid hatching asynchrony compared to its sister species. We studied hatching asynchrony and nestling provisioning in relation to food availability in sympatric populations of blue and great tits. For the first time, we show that sister species respond to food availability with different strategies. Blue tit feeding rates readily responded to the abundance of their main prey, and also reduced the impact of nestling size hierarchy on mean nestling weight, consequently increasing fledging rate. Our results suggest that levels of hatching asynchrony seem to be influenced by species-specific life history traits, as generalist foragers rely less on it. They also highlight the importance of multi-species approaches when studying the response of organisms to environmental unpredictability.

Selection intensity varies according to the phenology, and spatial pattern of food peak changes from year to year in a wide variety of ecosystems (reviewed for birds in ref. 1; see also refs 2 and 3). In the face of current climate change, breeding mismatches across trophic levels are arising in all ecosystem types, from terrestrial to marine habitats, threatening hundreds of well-established trophic interactions^4^. As individuals make decisions about breeding timing well before their offspring needs are at their highest, and because the optimal reproduction window is usually narrow^1^,^2^,^5^,^6^, species belonging to higher trophic levels must employ mechanisms to reduce mistiming. One of these mechanisms is the adjustment of breeding onset to prevailing environmental conditions, a strategy that can be fine-tuned. It has been shown, for instance, that laying date in great tits *Parus major* can be predicted by the phenology of the nearest oak to a nesting site^6^. Another mechanism is compensatory/delayed growth, as animals born late/early in the season can accelerate/delay their growth to correct their mistiming (reviewed for several taxa in ref. 7). In addition, to prompt hatching asynchrony (hereafter HA) has been proposed as a behavior to cope with environmental unpredictability in birds^8^. HA is defined as the time span between the hatching of the first and last egg within the same clutch, and in altricial birds it can last from a few hours to several days (reviewed in refs 9 and 10). As chicks are fed and gain weight from their first hours of life, a size gradient among them is established relative to HA^9^,^10^. The so-called ‘brood reduction hypothesis’ (hereafter BRH) suggests that HA is voluntary and adaptive when food is unpredictable^8^. Thus, birds would prefer to raise asynchronous broods that guarantee that at least larger nestlings fledge if food becomes scarce^8^,^11^,^12^. If environmental conditions ultimately allow for good provisioning rates, the whole brood will be successful as smaller nestlings will receive enough food^13^,^14^. A synchronous brood could compromise chick survival if conditions are ultimately disadvantageous^8^,^9^,^15^. Nevertheless, several alternative and, sometimes, non-exclusive hypotheses have been proposed to explain HA (reviewed in refs 9, 10 and 16). One of the most commonly accepted explanations is the ‘hurry-up hypothesis’ (hereafter HUH), which suggests that HA is a non-selected consequence of incubation starting before the clutch is complete, as a response to the ending of the breeding season (ref. 17; see also e.g., refs 16, 18 and 19). This behavior mismatches the incubation periods of the individual eggs, as the first eggs hatch earlier because they have been incubated for more days^20^,^21^,^22^,^23^.

Intra-population and interspecific variations in the levels of HA, together with its plasticity, are the least studied aspects of HA (ref. 10) found that there is a lack of non-experimental studies comparing different natural conditions (ref. 24) recommended the study of the variation of HA in relation to different, natural levels of food availability, as these have been rarely measured (but see refs 6, 12, 15 and 25; whereas other authors have studied proxies for food availability like bud burst or vegetation green up^3^,^19^). This is important because flexibility in food provisioning rates could be an alternative to HA in the best foragers (species or individuals). As food unpredictability is proposed as the reason behind the BRH^8^,^9^,^10^,^16^, those species (or individuals) that are better foragers could compensate for future food shortages, and would not need to resort to HA. It seems conceivable that high foraging efficiency is also adaptive to cope with food unpredictability, as HA without brood reduction can have long-lasting costs both for the female^16^,^26^, and for the smallest fledglings^27^,^28^.

Among birds, passerines present great flexibility in the HA levels^10^,^29^. Whereas annual or between-habitat variations in HA have been explored for single species^16^,^18^, interspecific responses (i.e., different species facing the same natural conditions) have not been studied. Tits are ideal species in which to study HA as they have a wide clutch size range (2–18), their clutches can hatch asynchronously^30^, and only females incubate. We studied HA in two sister species, the great and the blue tit *Cyanistes caeruleus*, in a sympatric population under natural conditions. Tits base their diets on Lepidoptera caterpillars^5^,^31^,^32^,^33^, with noctuids, tortricids and geometrids being the families primarily consumed in our study area^34^. However, noctuids are the preferred prey because they reach larger sizes. The higher their proportion in the nestling diet, the heavier the nestlings^5^. Thus, we expected that: (i) HA would be larger in those pairs mismatched with peak abundance, as the BRH suggests; (ii) following the HUH, there will be a positive relationship between HA and hatching date, because as caterpillar availability markedly decreases after the peak^5^, late breeders would benefit from starting the incubation before the clutch is complete; (iii) HA would predict the disparity in chick sizes within the brood, as initial differences in size would be maintained at fledging^16^,^19^,^35^; consequently, (iv) the larger the disparity in chick sizes (or HA), the lower the fledging rate, due to the starvation of the smallest chicks^18^,^19^, but see ref. 35; and finally, as blue tits are more generalist feeders than great tits^32^,^34^, but see ref. 33, we expect (v) blue tits to better track food availability, and, consequently, (vi) blue tits will rely less on HA to cope with environmental variability.

## Material and Methods

### Ethical statement

Catching and ringing protocols and the general ethic of our research was approved by the Junta de Comunidades de Castilla-La Mancha, Consejería de Agricultura y Medio Ambiente (licenses avp_11_1467, avp_12_061 and avp_13_059) in accordance with current Spanish laws.

### Study species

Blue and great tits have similar nesting biology as they lay one egg per day, with incubation periods of 13 days^30^. The great tit is larger (17–21 g) than the blue tit (7–11 g). The two species widely use nest-boxes provided for nesting (e.g., refs 19, 30 and 36). As noted above, both species forage on similar prey items.

### Study area

The present work was carried out during the 2012–2013 breeding seasons in San Pablo de los Montes (39°32′44″N, 4°19′41″W), a locality at Montes de Toledo (central Spain). Eight oak (*Quercus pyrenaica*)-dominated forests of different sizes (range 2.5–26.0 ha), and supplied with a proportional number of wooden nest-boxes (12.0 × 11.5 × 16.5 cm) were chosen (Table S1, Supporting Information). The nest-boxes were separated by 30–50 m from each other, and forest plots were at least 400 m apart. In our study region, deciduous woodlands are fragmented and alternate with scrublands, pasturelands, and other less suitable forests, like pinewood plantations^36^,^37^.

### General field protocol

We monitored 211 nests (92 in 2012, 119 in 2013), 135 (56 and 79, respectively) of them belonging to blue tits and 76 (36 and 40) to great tits. Clutches consisting of a single egg were excluded, although these were rare (<1%). Nests were visited daily from early April to mid-July. We assumed that the clutch size was complete when there was no increase in egg number after four days from the day the last egg was laid. We monitored hatching asynchrony in detail (see below). Eggs failing to hatch were those that were still present in the nest eight days after hatching (i.e., when we trapped the parents). Hence, if the brood size was lower than clutch size on these first days, missing eggs were assumed to have hatched, and the chicks concerned were assumed to have died, and to have been removed by the parents^36^. Parents were captured by means of spring traps when feeding nestlings 8–9 days old. At capture, adults were aged and identified with metal rings. The nestlings were banded and weighed when they were 13 days old. On day 22, nest-boxes were visited to assess the ‘fledging rate’, as chicks have already left the nest at this age^30^. Fledging rate is the proportion of chicks fledging to the number of chicks hatched, and it was only calculated for broods in which at least two chicks fledged. Second clutches (<1%) as well as mixed clutches (3%^36^) were excluded.

We surveyed the availability of noctuid, tortricid and geometrid caterpillars with the schema fully described in Table S2, based on that from^5^. We estimated the size of every single caterpillar with the ordinal scale described in ref. 38, and the biomass from the corresponding family was the sum of all the individuals from that family, which were used in the analyses as ‘noctuid availability’ or ‘tortricid availability’. We did not include the availability of geometrids in our analyses because this was much lower (Fig. S1, Supporting Information; see also below).

### Measuring HA

The manner in which HA is measured is a pivotal issue. For instance, the level of advantageousness that HA imparts in the case of the HUH depends on the time saved during incubation^9^,^10^. Because HA is commonly related to larger clutches (e.g., refs 12, 16, 18 and 25; but see ref. 19), it is important to control for clutch size. Additionally, the number of infertile eggs can affect the hatching hierarchy^15^. Thus, we followed the method described in ref. 15, in which ‘HA estimates’ (simply ‘HA’ hereafter) are the residuals from a lineal regression of brood size (number of chicks born) on hatching span (in days), assuming that all the eggs have a similar development period, and the laying of one egg per day. This implies that nests with a larger HA have more asynchronous broods relative to that expected for a brood of similar size^15^. This approach treats brood size as a continuous variable, which is inexact. However, this procedure does not affect results and facilitates the analysis, as sample size does not need to be stratified among the different clutch sizes^15^.

From the estimated day 11 of incubation, (i.e., two days before the expected hatching date), we carried out daily visits to record hatching date. The visits were ended after three consecutive days without new hatchings. To check the suitability of our method, we carried out two visits per day in a subset of 24 nests (12 great and 12 blue tit nests). In these nests, the first visit was before 12:00 P.M. (noon) and the second one after 5:00 P.M. We statistically compared the HA obtained with one visit *vs*. that with two visits per day, and confirmed that they resulted in similar estimates (Spearman correlation, R^2^ = 0.87, P < 0.0001). We thus used HA based on daily visits in all analyses and in all nests.

The relative weight difference (RWD) was used to describe the extent of the size hierarchy within broods [RWD = (heaviest − lightest nestling)/(mean nestling weight)]^39^; see also ref. 35). This was assessed when chicks were 13 days old.

### Diet filming

We filmed 124 nests (63 in 2012, 61 in 2013), 85 (45 and 40, respectively) of them belonging to blue tits and 39 (18 and 21) to great tits. When nestlings were 10 days old (i.e., 24 h before the filming day), we placed a wooden housing fixed to the back of the nest-box facing the entrance hole to allow birds to habituate to the future presence of the camera (Sony DCR-SR-type or HDR-XR550, all of them equipped with Night-shot system^TM^). When nestlings were 11 days, feeding was recorded with the video camera installed inside the housing, following the protocol described in refs 5 and 38. To control for filming ‘hour’, this variable was included in the analyses (see below). The first 60 minutes of each film were discarded to exclude potential disturbance effects on adult behavior from the camera placing. We studied the diet in the following 60 minutes as described in refs 5 and 38. Recordings were played and analysed frame-by-frame using the software Adobe Premiere Elements 7.0. We used a reference collection with prey sampled in the field to identify the images. Food items from a total of 3209 feeding trips were identified following categories defined in ref. 34. However, we focused on the larvae of two Lepidoptera families: tortricids and noctuids, as they formed >85% of the nestling diet in our study population^34^. They are used as a binomial of preferred *vs*. suboptimal prey by tits^5^,^34^. A scale (0–4 cm) was drawn inside the nest-box door to classify every prey into size categories. The variable ‘biomass of noctuids in diet’ used in our analyses was calculated as prey biomass per chick and hour. Thus, we multiplied prey sample size in every category by the ordinal categories of body size, summing all of them, and dividing the total by the number of nestlings (see also Table S2, Supporting Information). The same approach applies to ‘biomass of tortricids in diet’. Estimating biomass is a key step as tits select the largest rather than the most abundant prey^40^.

### Statistical analyses

We constructed general linear mixed models (GLMMs) with normal error and identity link, using PROC MIXED in SAS 9.0 (SAS Institute, Cary, NC, USA) to confirm the lack of differences in basic breeding parameters (laying date, hatching date and clutch size) between species and years, including their interactions.

We proceeded with Wilcoxon matched pairs and Student’s paired t tests when appropriate to analyze between-year differences in noctuid and tortricid biomasses within the eight forests and overall. These statistical analyses were performed with Statistica v.10 (Statsoft, Tulsa, OK, USA). Data are expressed as mean ± standard error (SE).

We constructed a GLMM (normal error and identity link) as implemented in the “lmer” procedure of the R package “lme4”^41^ to investigate which variables contributed to explaining the biomass of noctuids in the nestling diet. We included as categorical explanatory variables: ‘year’ (because 2012 and 2013 differed in caterpillar availability, see Results) and ‘species’ (blue tit *vs*. great tit). As continuous explanatory variables we used noctuid availability (the mean biomass of noctuids per survey in that forest on the date of filming), and filming hour. Forest identity was included as a random factor. Here and hereafter, we included the interaction terms between categorical variables, and between each categorical variable and each continuous explanatory variable. We used the best subset procedure and second-order AIC corrected for small sample sizes (AICc) to identify the set of models best explaining the biomass of noctuids in the nestling diet, as implemented in “dredge” and “model.avg” procedures of the R package “MuMIn”^42^. To evaluate the relative explanatory power of competing best models, we calculated Akaike weights (ωi). Here we present those model subsets whose weights sum ≥0.95. In order to estimate the relative importance of the variables included in the best models, we calculated the sum of Akaike weights of the models where these variables were included^43^. We repeated the same schema to evaluate the factors contributing to the biomass of tortricids in the nestling diet, but including tortricid availability as a continuous explanatory variable instead of noctuid availability.

To examine the role of different factors on HA we constructed a GLMM with the following categorical explanatory variables: year, species and ‘female age’ (yearling *vs*. adult; because female age could influence HA^25^). As continuous explanatory variables we included ‘hatching date’, ‘female mass’ (because smaller females could not effectively cover the whole clutch^19^,^44^) and both noctuid and tortricid availabilities. Forest and female identities were included as random factors.

To examine the role of different factors in RWD, we constructed a GLMM with year and species as categorical explanatory variables. As continuous explanatory variables we included HA and both noctuid and tortricid biomasses in the diet. Forest identity was again included as a random factor.

We evaluated the factors contributing to ‘mean nestling weight’ with a GLMM with year and species as categorical variables and HA, RWD and both noctuid and tortricid biomasses in the diet as continuous predictors. Forest identity was included as a random factor.

To analyze the factors influencing the fledging rate we assessed a GLMM with a binomial distribution and logit-link function as implemented in the “glmer” procedure of the R package “lme4”^41^. We included year and species as categorical variables and HA and RWD as continuous variables. Forest identity was included as a random factor.

## Results

### Basic breeding parameters

There were no differences between species in laying date (F_1,179_ = 1.60; P = 0.27), hatching date (F_1,179_ = 0.74; P = 0.43) or clutch size (F_1,179_ = 4.58; P = 0.08). There were also no differences between years in laying date (F_1,179_ = 1.62; P = 0.25), hatching date (F_1,179_ = 1.17; P = 0.33) or clutch size (F_1,179_ = 1.05; P = 0.39).

### Caterpillar availability

The mean biomass availability of noctuids per survey was 0.90 ± 0.16 in 2012 and 0.63 ± 0.10 in 2013, and although the pattern was consistent in 7 out of 8 forests (Table S2, Supporting Information), these differences were not significant (t = 1.70, P = 0.09). On the contrary, the mean availability of tortricids was significantly higher in 2013 (2012, 0.43 ± 0.07 *vs*. 2013, 1.23 ± 0.12; t = 7.10, P < 0.0001), and the pattern was highly consistent among forests (Table S2, Supporting Information). Geometrids were the least abundant family with 0.35 ± 0.08 in 2012 and 0.25 ± 0.06 in 2013 (t = 1.23, P = 0.22), but lacked a clear pattern (Table S2, Supporting Information). The shapes of the curves of caterpillar availability were variable among forests and between years, presenting two peaks, a single peak or even a plateau (Fig. S1, Supporting Information).

### Chick diet

Overall, we recorded a total of 3209 feeding trips, 73.6% of which were Lepidoptera larvae, accounting for 78.4% of the total biomass. Within Lepidoptera families, noctuids represented 63.7% and tortricids 15.2% of the total biomass. Geometrids only accounted for 6.6% of the total biomass.

**Figure 1:**
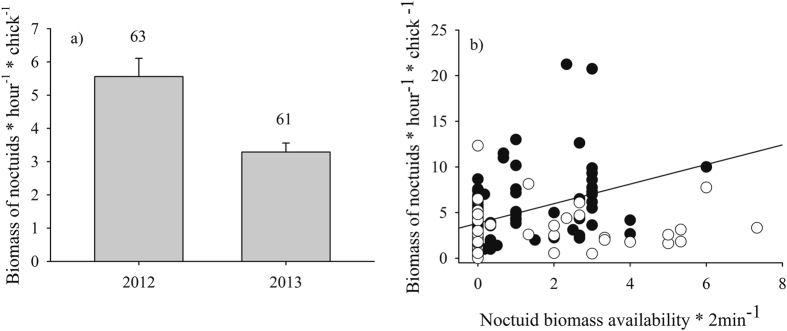
The biomass of noctuids in the nestling diet related to year (**a**) and to the availability of this family in the field when nestlings were 13 days old (**b**). In (**a**), the SE bars and samples sizes are shown. In (**b**), blue tits are represented by solid circles and by the regression line, and great tits by open circles.

The modelling approach provided a set of 21 models (whose weights summed ≥0.95) to explain the biomass of noctuids in the nestling diet (Table 1). Our results did not lend definitive support to any of these models due to the relatively small differences in AICc values (Table 1). Importantly, however, most models included the year (Σωi = 0.99), as the biomass of noctuids in nestling diet was greater in 2012 (Fig. 1a). Also influential were species (Σωi = 0.91) and noctuid availability (Σωi = 0.79). Nevertheless, their iteration was influential as well (Σωi = 0.45), as feeding with noctuids by blue tits was positively determined by their availability in the field, but this was not the case in great tits (Fig. 1b).

None of the potential predictor variables influenced nestling feeding with tortricids, as the null model was the one with the lowest AICc, not including any variable, and having a weight of 0.67 (Table S3, Supporting Information).

### Hatching asynchrony

**Figure 2:**
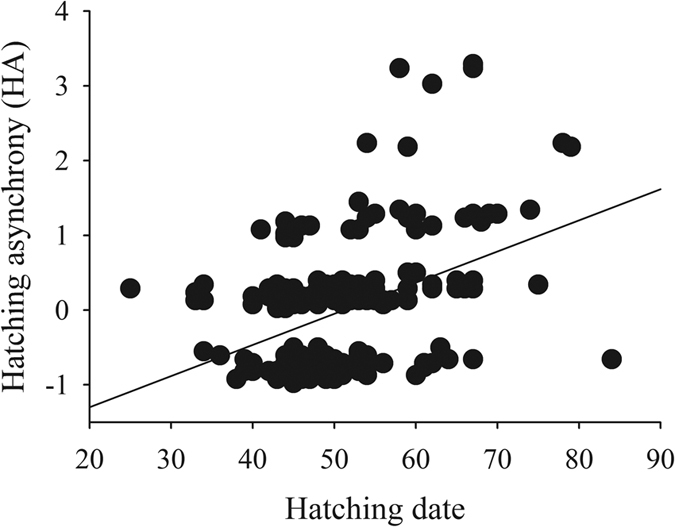
The hatching asynchrony related to the hatching date. The common regression line is also shown.

Hatching span varied from 1 (all the chicks hatched on the same day) to 5 days in both species. Model building selected a subset of 5 models (Table 2), being hatching date the only influential variable, but present in all the models (Σωi = 1.00). The later the hatching date, the larger the HA (Fig. 2).

### Relative weight difference

**Figure 3:**
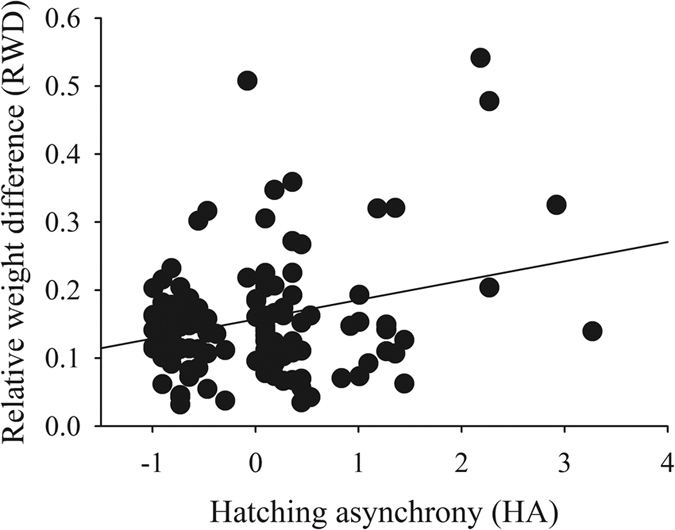
The relative weight difference related to hatching asynchrony. The common regression line is also shown.

Only one model was selected (Table S4, Supporting Information), as the null model was the second best. HA was the single variable included in this model (ωi = 1.00), and it positively influenced the RWD (Fig. 3).

### Mean nestling weight

**Figure 4:**
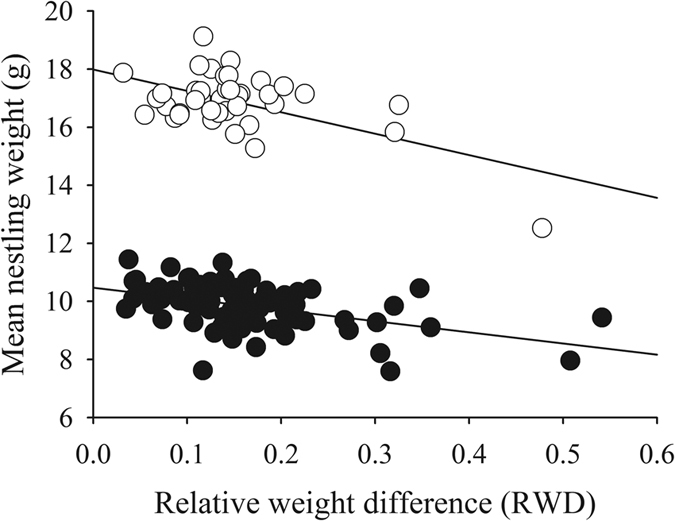
The mean nestling weight related to the relative weight difference. Blue tits are represented by solid circles, and great tits by open circles. Regression lines are also shown.

Model building generated a subset of 29 models (Table 3). As expected due to the differences in species size, species was a highly influencing variable in mean nestling weight, being present in all the models (Σωi = 1.00). Year was present in all the models as well, because nestlings were heavier in 2013 (Fig. S2, Supporting Information). Also, RWD was present in all the models; however, its interaction with species was highly influential (Σωi = 0.91), as the larger the RWD, the lower the mean weight, but being more pronounced in the case of great tits (Fig. 4).

### Fledging rate

**Figure 5:**
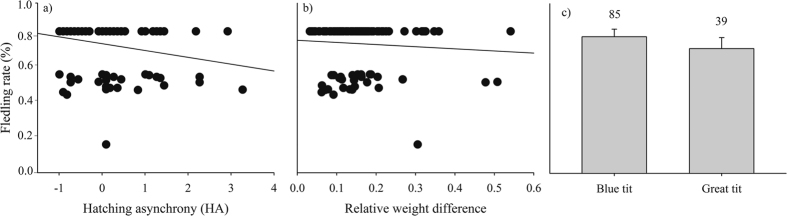
The fledging rate related to hatching asynchrony (**a**), relative weight difference (**b**) and species (**c**). Common regression lines are shown in (**a**) and (**b**). In (**c**), the SE bars and samples sizes are shown.

Modelling procedure generated a subset of 37 models to explain fledging rate (Table 4). Due to their small differences in AICc values, our results did not lend definitive support to any of them. Nevertheless, the variable year was present in all the models (Σωi = 1.00), as fledging rate was higher in 2013 (Fig. S3, Supporting Information). Influential were also HA (Σωi = 0.88) and RWD (Σωi = 0.62) as both of them negatively affected fledging rate (Fig. 5a,b). Finally, species was influential as well (Σωi = 0.70), having blue tits larger fledging rates than great tits (Fig. 5c).

## Discussion

By comparing two sympatric species, our findings suggest that differences previously found in HA levels in comparative reviews of single-species studies^9^,^10^,^29^,^45^ could be related to more generalist or more specialist foraging strategies of the studied species. Namely, specialists like great tits would rely more intensely on HA, whereas more generalist birds, such as blue tits, would take advantage of their flexible feeding capabilities to cope with food unpredictability, thus avoiding HA. Our results should be interpreted with some caution as trophic generalist species may include specialized individuals consistently feeding on certain resources^46^.

Ecosystems are often highly variable from one year to another, and species with short life expectancies, such as small passerines, must adjust their breeding timing to these unstable conditions^3^,^6^. These species need mechanisms to cope with environmental unpredictability, especially with respect to changes in food availability, which determines their breeding performance^5^,^31^,^40^. The variety of shapes in the curves of caterpillar availability in our study highlights both the temporal and spatial unpredictability of food availability that tits must face. For instance, our study area underwent unusual snowfalls at the beginning of May 2013, which stopped leaf development, and, in turn, modified caterpillar availability. One mechanism that has been proposed for birds to cope with this uncertainty is HA. HA creates a hierarchy of sizes among the nestlings that ensures the survival of larger nestlings when food is running short, while smaller nestlings die, rather than compromising the survival of the whole brood^8^,^12^. However, smaller nestlings can also survive if food provisioning is ultimately sufficient^13^,^14^. Our results suggest that foraging efficiency is a suitable alternative to HA as those species (or individuals) that are better foragers could compensate for future food shortages, and would not need to resort to HA. Blue tits were more efficient foragers because they consistently increased the biomass of noctuids in the nestling diet when these prey were more available in the field, whereas great tits did not. Also, RWD caused a greater impact on mean nestling weight in great tits (i.e., their broods were more heterogeneous, lowering the smallest chicks the overall mean), showing lower fledging rates. In other words, blue tits, but not great tits, showed compensatory feeding, thus minimizing the impact of nestling size hierarchy produced by HA. In a previous study, we found that blue tits, but not great tits, showed compensatory investment in larger broods by increasing the feeding rate with suboptimal prey^34^. Indeed, blue tits fed with smaller prey, more frequently, and with higher prey diversity^34^, see also ref. 32. All of these traits correspond to a generalist foraging strategy. Monitoring feeding rates was key to determining how blue tits compensated for HA, as some experiments that tested the role of food provisioning in HA stopped the supplementary feeding before incubation started (e.g., refs 12 and 15). Plastic responses are vital when evolution is insufficient to keep track of environmental changes, and can help species to counter stressful conditions^47^. Our results suggest that both HA and compensatory feeding can be suitable responses to the new selection pressures originated by climate change. As commented above, the existence of unstable environmental conditions is one of the reasons suggested to be behind HA^8^,^11^,^12^. On the other hand, it is widely assumed that ecological generalists are less vulnerable to environmental changes, and less affected by changes in resources availability compared to their specialist counterparts^48^. This is the case of the response of Darwin’s tree finches (*Camarhynchus spp*.) to dry years (i.e., conditions of resource scarcity), which varied with their trophic status, as generalist species were even more generalized during periods of food shortage, while the specialist species become even more specialized^49^. Also, penguin species with broader foraging niche has been found to respond better (i.e., population increase) to rapidly changing environmental conditions than their sister species with narrower niche (i.e., population decrease) in sympatric populations^50^. Finally, climate change is occurring at a time when forests are becoming increasingly fragmented through habitat destruction^47^, and individuals occupying suboptimal patches could evolve in a particular ways (e.g., being smaller^51^). These could be interesting topics for future studies.

Our results suggest that great tits likely resort to HA because they are not able to improve their feeding efficiency to respond to environmental variability as blue tits do (see also refs 32 and 34). However, the foraging flexibility shown by the latter could be not enough at the end of season, when caterpillar availability diminishes because they pupate^5^,^6^. The increase in HA as the season advanced was a common pattern in several studies, based on the reduction of resource availability with date^16^,^17^,^18^,^19^. This pattern aligns with the expectations of both the BRH and the HUH, as the costs of synchrony are particularly high under the poor environmental conditions that typically affect late broods, reducing nestling survival^15^,^16^,^19^.

The response to food availability was, however, similar between blue and great tits in some respects. The biomass of noctuids delivered to the nestlings was more than four times that of tortricids. Noctuid caterpillars are larger, juicy, allow for fewer feeding trips, and are related to higher nestling weight^5^. This makes them ideal prey for central place foragers like tits, as birds can maximize foraging rates by reducing the trips to and from a nest. On the contrary, tits did not track tortricid availability, as this did not influence the delivery rates of caterpillars of this family to the nestlings, even during the 2013 outbreak, when tortricid abundance was three times that of 2012, and noctuid abundance was low. This underrepresentation of tortricids in the nestling diet has previously been observed in other Mediterranean oak woods, and seems related to their inferior nutritional quality^5^.

The hierarchies caused by HA were maintained throughout the whole nestling period, as found in other works^12^,^19^,^52^. The influence of tortricids in nestling parameters was limited. Although nestling mean weight as well as fledging rate increased during the tortricid outbreak in 2013, tits were not able to compensate for the size gradient due to the HA simply by increasing the feeding rate with these suboptimal prey. Consequently, the HA (and the RWD) negatively affected the fledging rate^18^,^19^, but see refs 16 and 35. Despite nestling mortality was low (overall 8% in blue tits and 7% in great tits; lower than in other studies with tits: 19–21%^16^ or 16–22%^53^; see also ref. 35), those nestlings that died were likely the smallest ones^18^. The reason for the lower nestling mortality in our populations could be their smaller brood sizes compared with those studies from northern populations. This, in turn, seems to be related to the low egg hatchability due to understory overgrazing, what could affect the abundance of snails, the main source of calcium for the eggshell in our study region^54^. Although it could be interesting to repeat our approach with higher levels of chick mortality, we are confident that these do not constrain our findings, as our sympatric species showed similar levels of nesting mortality but presented different strategies (HA *vs*. foraging efficiency).

Some authors^19^ have suggested that those blue tits with larger HA were late breeders, and altogether worse birds (younger, worse competitors), implying that HA is not deliberate. However, female age did not influence HA in our study population (the same applies to great tits), thus not supporting the idea that HA is linked to suboptimal females (see also ref. 21; but see ref. 53). Nevertheless, we acknowledge that we only discriminated two age classes. Thus, these findings should be treated with caution. In their study with pied flycatchers *Fycedula hypoleuca*^11^, these authors found that yearling females did hatch more synchronously, which they interpreted as a result of energy constraints occurring at laying time^18^. The asynchronous hatching in yearling Eurasian kestrels *Falco tinnunculus* has been suggested as a voluntary adjustment to their lower ability for food provisioning^25^. Finally, female mass did not influence our results, rejecting the idea that the incapacity to manage large broods^19^,^41^ plays a role in our results.

If HA is adaptive, it should lead to heavier nestlings, reducing fledging rate due to the mortality of smaller chicks (the ‘offspring quality assurance hypothesis’^16^; see also ref. 18). However, in our study, HA reduced fledging rates in both species, but did not increase mean body weight, but rather quite the contrary. Our results showed that RWD (closely related to HA) reduced the mean nestling weight, mainly in great tits. In enlarged great tit broods^35^, found similar results, as did^55^ after manipulating HA in Eurasian kestrels, although only in food-limited years. In our study years, caterpillars were less abundant than, for instance, in 2011 (unpub. data). Despite the poor environmental conditions, many small nestlings survived, which surely reduced brood mean weight. The body mass at fledging positively predicts post-fledging survival and recruitment in tits^16^,^56^. Despite the fact that HA does not seem to be adaptive for fledglings, it may still be adaptive for their parents if: (i) with the progression of the season, this best-of-a-bad-job provides higher fitness than losing the entire brood due to trying to get ahead with synchronic fledglings when food availability decreases^8^,^12^,^18^,^19^; (ii) chicks die earlier in asynchronous than in synchronous broods, and thus parents can save energy expenditures not feeding chicks that are likely to die anyway^9^,^19^, but see ref. 52; or, (iii) HA reduces year-to-year variance in parental fitness via offspring recruitment^53^. Finally, we cannot discard the idea that some physiological, environmental or other factors constrained incubation behavior, and thus influenced HA^18^,^19^,^57^. Once nestlings hatched, other constraints like food allocation among nestlings of different sizes could also influence their growth and mortality^58^.

Several sources of evidence support our findings that blue tits are opportunistic species, with a better ability to track environmental variability than great tits: (i) blue tits were more synchronized with vegetation green-up and more strongly influenced by local habitat quality than great tits^3^; as noted above (ii), blue tits showed generalist feeding habits, with a higher feeding rate, providing smaller prey, and with higher prey diversity than great tits^32^,^34^; (iii) seasonal movements are a major component of opportunistic species population dynamics, easily responding to environmental fluctuations^59^. Blue tits, but not great tits, responded to warmer winters by decreasing their displacement distances, as closer proximity to the breeding grounds in winter allows better prediction of the spring onset; blue tits also showed a stronger advancement of laying date than great tits^60^; (iv) age of first reproduction is earlier in generalist species, or, in other words, specialists postpone their reproduction^59^,^61^. Clutch size, nesting success, brood size and juvenile survival all decreased earlier in blue tits than in great tits; that is, females became old earlier in the former species^62^; (v) generalists do not compete and do not become dominant^59^,^61^. In a previous work, we showed that great tits outcompeted blue tits for nest-holes, with some aggressions even leading to blue tit death^36^.

In sum, our study explored for the first time the natural levels of HA in two sympatric species in relation to food abundance and nestling provisioning. Our findings add to the evidence that there is no single pressure influencing HA in all bird species, as patterns of HA –and, consequently, nestling size hierarchy or fledging rate - differed depending on the degree of specialist/generalist feeding at the species level. They also highlight the importance of simultaneously studying more than one species when analyzing the response of animal populations to a shared, variable environment.

## Additional Information

**How to cite this article**: Barrientos, R. *et al*. Hatching asynchrony *vs*. foraging efficiency: the response to food availability in specialist *vs*. generalist tit species. *Sci. Rep.* **6**, 37750; doi: 10.1038/srep37750 (2016).

**Publisher's note:** Springer Nature remains neutral with regard to jurisdictional claims in published maps and institutional affiliations.

## Supplementary Material

Supplementary Material

## Figures and Tables

**Table 1:** Subset of models explaining the biomass of noctuids in nestling diet.

Biomass of noctuids in nestling diet	Estimates									AICc	Weight
	Year	Hour	Noctuid availability	Species	Year: hour	Year: noctuid availability	Year: species	hour: species	Noctuid availability: species		
Model 1	+		0.468	+					+	658.7	0.18
Model 2	+		0.436	+						659.4	0.13
Model 3	+		0.412	+			+		+	659.8	0.11
Model 4	+		0.379	+			+			660.4	0.08
Model 5	+			+			+			660.6	0.07
Model 6	+			+						661.1	0.06
Model 7	+		0.473	+	+				+	661.9	0.04
Model 8	+		0.361							662.1	0.04
Model 9	+									662.1	0.03
Model 10	+	0.090	0.456	+					+	662.2	0.03
Model 11	+	0.061	0.427	+						662.9	0.02
Model 12	+		0.437	+	+					662.9	0.02
Model 13	+		0.421	+	+		+		+	663.3	0.02
Model 14	+	0.088	0.400	+			+		+	663.3	0.02
Model 15	+	0.233	0.406	+				+	+	663.3	0.02
Model 16	+	0.118		+			+			663.8	0.01
Model 17	+	0.058	0.370	+			+			663.9	0.01
Model 18	+		0.381	+	+		+			664.1	0.01
Model 19	+	0.134		+						664.2	0.01
Model 20			0.526	+					+	664.3	0.01
Model 21	+	0.150								665.0	0.01
	**Multimodel Inference**										
**Σωi**	0.99	0.19	0.79	0.91	0.01	0.11	0.35	0.05	0.45		
**Weighted average β**	1.00	0.12	0.43	0.68	−0.18	−0.11	0.46	−0.31	0.40		
**SE β**	0.45	0.22	0.19	1.06	0.21	0.20	0.34	0.23	0.19		

Only models summing ≥0.95 of AIC weights are shown. AIC weight is the estimated probability that a model is the best model in the set. Multimodel inference has been obtained considering all the possible combinations of predictors, averaging the results according model weights (ωi). For each variable, Σωi is the sum of weights of the models in which the variable appears, weighted average β is the weighted average of conditional adjusted regression coefficients, and SE β the conditional adjusted standard errors.

**Table 2:** Subset of models explaining the hatching asynchrony.

Hatching asynchrony	Estimates							AICc	Weight
	Female age	Female mass	Year	Hatching date	Noctuid availability	Species	Tortricid availability		
Model 1				0.054				480.5	0.74
Model 2				0.050	−0.046			485.2	0.07
Model 3				0.055		+		485.6	0.06
Model 4	+			0.055				485.7	0.06
Model 5			+	0.054				486.4	0.04
	**Multimodel Inference**								
**Σωi**	0.05	0.01	0.04	1.00	0.07	0.06	0.03		
**Weighted average β**	−0.05	0.01	0.03	0.05	−0.05	−0.05	−0.02		
**SE β**	0.05	0.01	0.05	0.01	0.03	0.05	0.05		

Only models summing ≥0.95 of AIC weights are shown. AIC weight is the estimated probability that a model is the best model in the set. Multimodel inference has been obtained considering all the possible combinations of predictors, averaging the results according model weights (ωi). For each variable, Σωi is the sum of weights of the models in which the variable appears, weighted average β is the weighted average of conditional adjusted regression coefficients, and SE β the conditional adjusted standard errors.

**Table 3:** Subset of models explaining the mean nestling weight.

Mean nestling weight	Estimates												AICc	Weight
	Biom. noctuids	Biom. tortricid	Year	HA	RWD	Species	Biom.Tort: Year	Biom.Tort: Species	Year: HA	Year: RWD	Year: Species	RWD: Species		
Model 1			+		−5.877	+						+	278.4	0.28
Model 2			+		−5.879	+				+		+	279.2	0.18
Model 3		−0.101	+		−5.635	+		+				+	280.1	0.12
Model 4		−0.102	+		−5.632	+		+		+		+	281.1	0.07
Model 5			+	−0.091	−5.522	+						+	282.6	0.04
Model 6			+		−6.046	+					+	+	283.2	0.03
Model 7			+	−0.092	−5.521	+				+		+	283.4	0.02
Model 8			+		−5.097	+				+			283.6	0.02
Model 9			+		−4.879	+							283.9	0.02
Model 10		−0.108	+	−0.100	−5.243	+		+				+	284	0.02
Model 11			+		−6.046	+				+	+	+	284.1	0.02
Model 12			+	−0.086	−5.343	+			+			+	284.4	0.01
Model 13		−0.020	+		−5.871	+						+	284.5	0.01
Model 14		−0.100	+		−5.803	+		+			+	+	285.1	0.01
Model 15		−0.108	+	−0.100	−5.243	+		+		+		+	285.1	0.01
Model 16		−0.116	+		−4.686	+		+					285.3	0.01
Model 17			+	−0.085	−5.340	+			+	+		+	285.3	0.01
Model 18		−0.099	+	−0.094	−5.054	+		+	+			+	285.4	0.01
Model 19		−0.020	+		−5.872	+				+		+	285.4	0.01
Model 20	−0.021		+		−5.895	+						+	285.5	0.01
Model 21		−0.107	+		−4.856	+		+		+			285.6	0.01
Model 22			+	−0.106	−4.652	+			+				286.1	0.01
Model 23	−0.030	−0.082	+		−5.654	+		+				+	286.1	0.01
Model 24		−0.101	+		−5.801	+		+		+	+	+	286.1	0.01
Model 25	−0.022		+		−5.903	+				+		+	286.3	0.01
Model 26		−0.102	+		−5.550	+	+	+				+	286.4	0.01
Model 27		−0.101	+	−0.092	−5.046	+		+	+	+		+	286.4	0.01
Model 28		−0.106	+	−0.113	−4.429	+		+	+				286.5	0.01
Model 29			+	−0.124	−4.731	+				+			286.6	0.01
	**Multimodel Inference**													
**Σωi**	0.02	0.32	1.00	0.18	1.00	1.00	0.01	0.30	0.06	0.39	0.07	0.91		
**Weighted average β**	−0.03	−0.10	−0.26	−0.10	−5.66	−3.83	0.03	0.18	0.16	0.13	0.07	1.99		
**SE β**	0.02	0.06	0.10	0.08	0.89	0.19	0.05	0.06	0.08	0.84	0.07	0.88		

Only models summing ≥0.95 of AIC weights are shown. AIC weight is the estimated probability that a model is the best model in the set. Multimodel inference has been obtained considering all the possible combinations of predictors, averaging the results according model weights (ωi). For each variable, Σωi is the sum of weights of the models in which the variable appears, weighted average β is the weighted average of conditional adjusted regression coefficients, and SE β the conditional adjusted standard errors.

**Table 4:** Subset of models explaining the fledging rate.

Fledging rate	Estimates									AICc	Weight
	Year	HA	RWD	Species	Year: HA	Year: RWD	Year: Species	HA: Species	RWD: Species		
Model 1	+	−0.155								477.9	0.06
Model 2	+	−0.214	1.269	+						478.0	0.06
Model 3	+	−0.172		+						478.0	0.06
Model 4	+	−0.191	1.116							478.3	0.05
Model 5	+	−0.192		+	+					478.5	0.05
Model 6	+	−0.233	1.226	+	+					478.7	0.04
Model 7	+	−0.208	1.406	+			+			478.7	0.04
Model 8	+	−0.168			+					478.9	0.04
Model 9	+									479.0	0.03
Model 10	+	−0.198	1.771			+				479.1	0.03
Model 11	+	−0.162		+			+			479.2	0.03
Model 12	+	−0.245	1.930	+	+	+				479.2	0.03
Model 13	+	−0.224	1.940		+	+				479.3	0.03
Model 14	+	−0.215	1.772	+		+				479.4	0.03
Model 15	+	−0.203	1.084		+					479.5	0.03
Model 16	+	−0.225	1.357	+	+		+			479.7	0.02
Model 17	+			+						479.7	0.02
Model 18	+	−0.181		+	+		+			479.9	0.02
Model 19	+	−0.211	1.916	+		+	+			480.1	0.02
Model 20	+	−0.211	1.164	+					+	480.2	0.02
Model 21	+	−0.169		+				+		480.2	0.02
Model 22	+	−0.214	1.271	+				+		480.3	0.02
Model 23	+	−0.239	2.066	+	+	+	+			480.3	0.02
Model 24	+			+			+			480.4	0.02
Model 25	+	−0.178		+	+			+		480.4	0.02
Model 26	+		0.610							480.6	0.02
Model 27	+	−0.221	1.188	+	+			+		480.8	0.01
Model 28	+	−0.239	1.417	+	+				+	480.9	0.01
Model 29	+	−0.206	1.402	+			+	+		481.0	0.01
Model 30	+	−0.209	1.448	+			+		+	481.0	0.01
Model 31	+		0.695	+						481.2	0.01
Model 32	+	−0.206	1.460	+		+			+	481.4	0.01
Model 33	+	−0.158		+			+	+		481.4	0.01
Model 34	+	−0.240	1.794	+	+	+			+	481.5	0.01
Model 35	+	−0.243	1.915	+	+	+		+		481.5	0.01
Model 36	+		0.840	+			+			481.6	0.01
Model 37	+	−0.228	1.853	+		+		+		481.6	0.01
	**Multimodel Inference**										
**Σωi**	1.00	0.88	0.62	0.70	0.37	0.22	0.26	0.14	0.09		
**Weighted average β**	−0.40	−0.20	1.44	−0.13	0.12	−1.21	−0.10	−0.02	0.10		
**SE β**	0.13	0.10	1.03	0.11	0.09	1.06	0.09	0.10	1.19		

Only models summing ≥0.95 of AIC weights are shown. AIC weight is the estimated probability that a model is the best model in the set. Multimodel inference has been obtained considering all the possible combinations of predictors, averaging the results according model weights (ωi). For each variable, Σωi is the sum of weights of the models in which the variable appears, weighted average β is the weighted average of conditional adjusted regression coefficients, and SE β the conditional adjusted standard errors.
