# Supplementary material for: Hatching asynchrony vs. foraging efficiency: the response to food availability in specialist vs. generalist tit species
Source: Sci Rep. 2016 Nov 28;6:37750. doi: 10.1038/srep37750 (PMC5125266; doi:10.1038/srep37750)

Article title: **Hatching asynchrony *vs.* foraging efficiency: the response to food availability in specialist *vs.* generalist tit species**

Journal title: **Scientific Reports**

Author names: **Rafael Barrientos [1,*], Javier Bueno-Enciso[1], Juan José Sanz[2]**

Affiliations:

[1] Área de Zoología, Departamento de Ciencias Ambientales, Facultad de Ciencias del Medio Ambiente, Universidad de Castilla-La Mancha, Avenida Carlos III, s/n, E-45071, Toledo, Spain.

* Corresponding author current addresses: 1) Infraestruturas de Portugal Biodiversity Chair, CIBIO/InBio, Centro de Investigação em Biodiversidade e Recursos Genéticos, Universidade do Porto. Campus Agrário de Vairão, Vairão, Portugal; 2) CEABN/InBio, Centro de Ecologia Aplicada "Professor Baeta Neves", Instituto Superior de Agronomia, Universidade de Lisboa, Tapada da Ajuda, 1349-017 Lisboa, Portugal

Email: barrientos@cibio.up.pt

[2] Departamento de Ecología Evolutiva, Museo Nacional de Ciencias Naturales (MNCN-CSIC), José Gutiérrez Abascal, 2, E-28006, Madrid, Spain.

Table S1.- Attributes for the plots monitored during the present study.

Table S2.- Mean caterpillar availabilities.

Table S3.- Subset of models explaining the biomass of tortricids in nestling diet.

Table S4.- Subset of models explaining the nestling relative weight difference.

Figure S1.- Caterpillar abundances along the season in both years.

Figure S2.- Mean nestling weight per species and year.

Figure S3.- Mean fledging rate per year.

Table S1.- Attributes for the eight forests monitored during the present study, including the number and the name of the study plot, the number of nest-boxes (No. nest-boxes) and the area (ha).

Study forest	No. nest-boxes	Area (ha)
“Baños”	10	2.5
“Casillas”	40	10.0
“Ermita”	10	3.6
“Lanchar”	20	7.0
“Majadillas”	40	8.4
“Marchés”	80	26.0
“Morra”	80	17.9
“Robledillo”	10	3.9

Table S2.- Mean biomass ± SE per survey for the three main Lepidoptera families in our study area. Outcomes from Wilcoxon and t-Student paired-sample tests are shown. Survey schema is explained in the footnote*. Significant P-values at α= 0.05 are highlighted in red.

	**Tortricids**			**Noctuids**			**Geometrids**		
Forest	2012	2013	Test	2012	2013	Test	2012	2013	Test
Baños	0.58±0.26	1.15±0.32	Z= 2.48, P= 0.01	1.42±0.65	1.02±0.32	Z= 0.31, P= 0.76	0.33±0.20	0.42±0.14	Z= 0.18, P= 0.86
Casillas	0.73±0.35	1.48±0.42	Z= 1.11, P= 0.27	0.58±0.21	0.23±0.12	Z= 1.24, P= 0.21	0.35±0.16	0.02±0.02	Z= 1.65, P= 0.10
Ermita	0.67±0.24	1.29±0.35	Z= 1.73, P= 0.08	1.31±0.73	0.71±0.30	Z= 0.28, P= 0.78	0.65±0.29	0.73±0.32	Z= 0.26, P= 0.80
Lanchar	0.27±0.10	1.17±0.35	Z= 2.77, P< 0.01	0.48±0.24	0.60±0.27	Z= 0.62, P= 0.53	0.04±0.04	0.25±0.14	Z= 1.27, P= 0.21
Majadillas	0.06±0.05	1.17±0.31	Z= 2.97, P< 0.01	0.83±0.56	0.60±0.30	Z= 0.65, P= 0.52	0.69±0.38	0.27±0.14	Z= 0.34, P= 0.73
Marchés	0.25±0.18	0.75±0.19	Z= 2.30, P= 0.02	0.64±0.29	0.60±0.34	Z= 0.41, P= 0.68	0.21±0.09	0.06±0.06	Z= 1.17, P= 0.24
Morra	0.46±0.20	1.19±0.39	Z= 1.19, P= 0.23	1.06±0.43	0.52±0.29	Z= 0.80, P= 0.42	0.48±0.25	0.15±0.08	Z= 0.85, P= 0.39
Robledillo	0.46±0.15	1.67±0.38	Z= 3.41, P< 0.001	0.90±0.31	0.75±0.29	Z= 0.83, P= 0.41	0.04±0.03	0.10±0.08	Z= 0.17, P= 0.86
**Mean**	0.43±0.07	1.23±0.12	t= 7.10, P< 0.0001	0.90±0.16	0.63±0.10	t= 1.70, P= 0.09	0.35±0.08	0.25±0.06	t= 1.23, P= 0.22

[*]Caterpillar availability throughout the breeding seasons was monitored in the eight forests by means of two surveys per week, from day 26-27 (1= 1st April) to 79-80 in both years following the method described in [1]. Every survey consisted of three different sampling points (i.e., mature oaks), dispersed across the forest. Thus, we carried out 16 surveys, totaling 48 sampling points, per forest and year. In every sampling point, we counted the Lepidoptera larvae from noctuids, tortricids and geometrids during a 2-min period. We used 2 minutes instead of 5 described in [1] because a pilot study provided similar results with both time lapses (data not shown). We also estimated the size of every caterpillar with the ordinal scale described in [2] to assess the total caterpillar biomass (i.e., 'availability'). Namely, 1 = small (body size length < 1.5cm), 2 = medium (length 1.5-2.5 cm), 3 = large (length 2.5-3.5 cm) and 4 = extra large (length> 3.5cm). We multiplied the total number of caterpillars of every size by that size value to obtain the biomass of that size category. Total biomass availability was the sum of the biomasses of the four categories. We averaged the three sampling points to obtain a single value per survey. In our study region, this method seems more accurate than the collection of caterpillar droppings[1]. Our method also allows caterpillar identification at the family level.

References

1. García-Navas, V. & J. J. Sanz. The importance of a main dish: nestling diet and foraging behaviour in Mediterranean blue tits in relation to prey phenology. *Oecologia* **165,** 639-649 (2011).

2. García-Navas, V. & J. J. Sanz. Flexibility in the foraging behaviour of blue tits in response to short-term manipulations of brood size. *Ethology* **116,** 744-754 (2010).

Table S3.- Subset of models explaining the biomass of tortricids in nestling diet. Only models summing ≥ 0.95 of AIC weights are shown. AIC weight is the estimated probability that a model is the best model in the set. Multimodel inference has been obtained considering all the possible combinations of predictors, averaging the results according model weights (ωi). For each variable, Σωi is the sum of weights of the models in which the variable appears, weighted average β is the weighted average of conditional adjusted regression coefficients, and SE β the conditional adjusted standard errors.

	Estimates					
**Biomass of tortricids in diet**	Year	Hour	Species	Tortricid availability	AICc	Weight
Model 1					401.1	0.67
Model 2				-0.112	404.6	0.12
Model 3			+		405.5	0.08
Model 4		-0.071			405.7	0.07
Model 5	+				405.9	0.06
	**MULTIMODEL INFERENCE**					
**Σωi**	0.06	0.07	0.09	0.13		
**Weighted average β**	-0.00	-0.07	-0.06	-0.11		
**SE β**	0.11	0.07	0.12	0.09		

Table S4.- Subset of models explaining the nestling relative weight difference. Only models summing ≥ 0.95 of AIC weights are shown. AIC weight is the estimated probability that a model is the best model in the set. Multimodel inference has been obtained considering all the possible combinations of predictors, averaging the results according model weights (ωi). For each variable, Σωi is the sum of weights of the models in which the variable appears, weighted average β is the weighted average of conditional adjusted regression coefficients, and SE β the conditional adjusted standard errors.

	Estimates		
**Relative weight difference**	HA	AICc	Weight
Model 1	0.029	-239.2	1.00
	**MULTIMODEL INFERENCE**		
**Σωi**	1.00		
**Weighted average β**	0.03		
**SE β**	0.01		

Fig. S1.- Mean biomass per survey for tortricids 2012 and 2013 (a, b), noctuids 2012 and 2013 (c, d) and geometrids 2012 and 2013 (e, f).

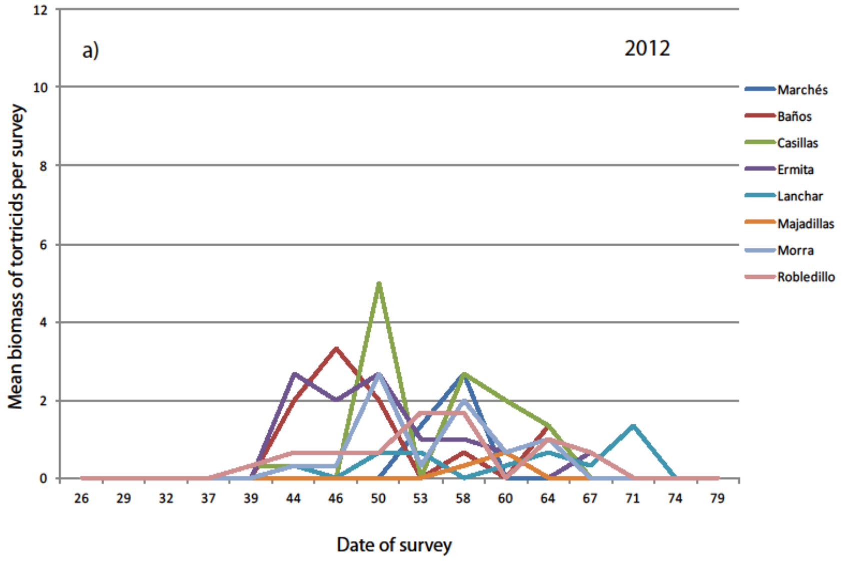

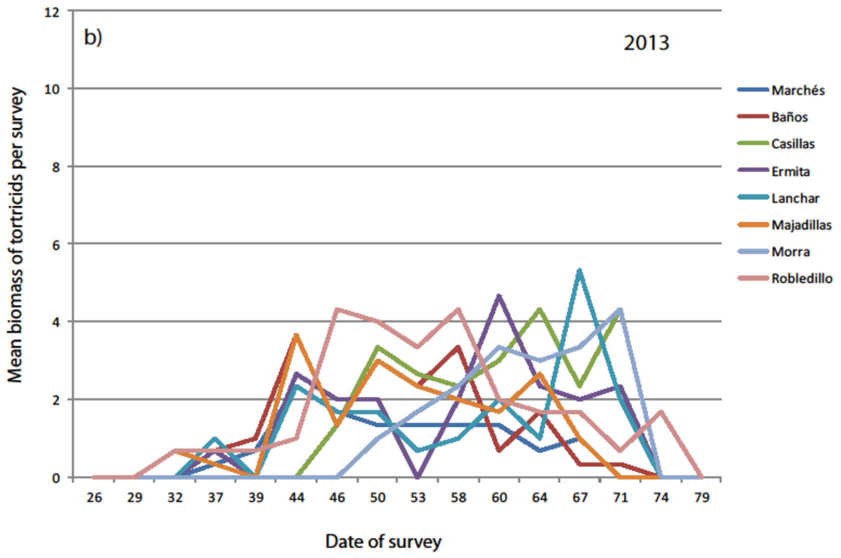

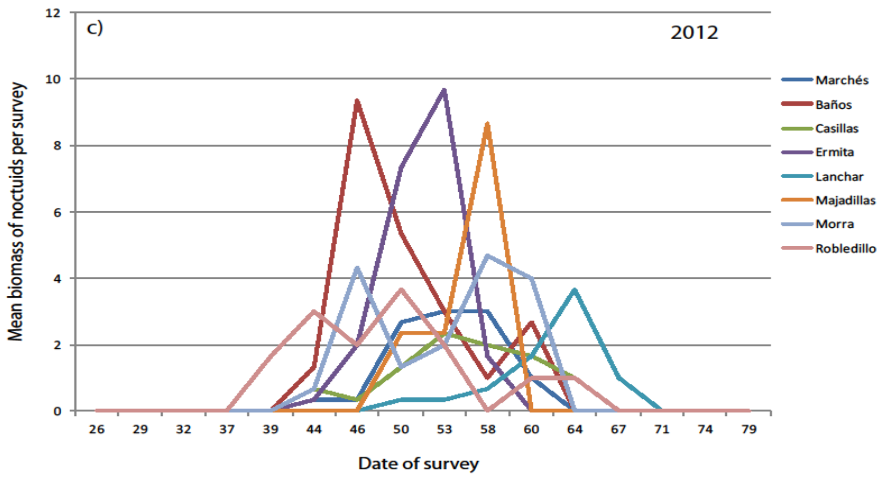

c)
2012
Mean biomass of noctuids per survey
Date of survey
0
2
4
6
8
10
12
26
29
32
37
39
44
46
50
53
58
60
64
67
71
74
79
Marchés
Baños
Casillas
Ermita
Lanchar
Majadillas
Morra
Robledillo

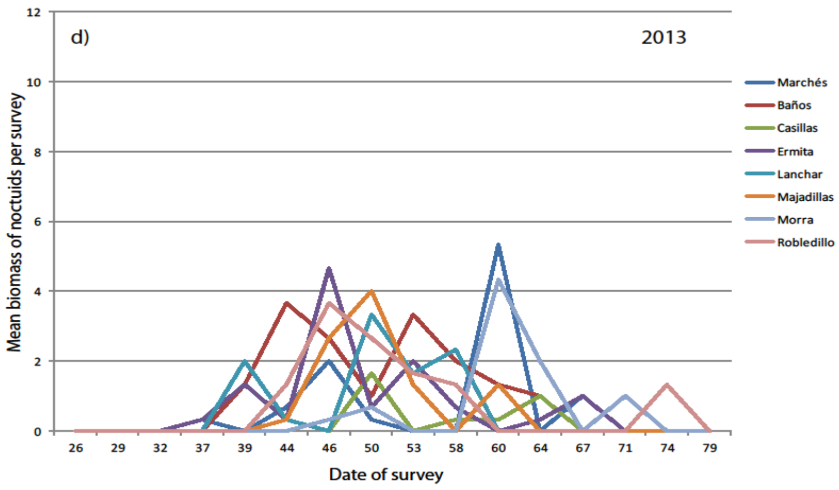

d)
2013
Mean biomass of noctuids per survey
Date of survey
0
2
4
6
8
10
12
26
29
32
37
39
44
46
50
53
58
60
64
67
71
74
79
Marchés
Baños
Casillas
Ermita
Lanchar
Majadillas
Morra
Robledillo

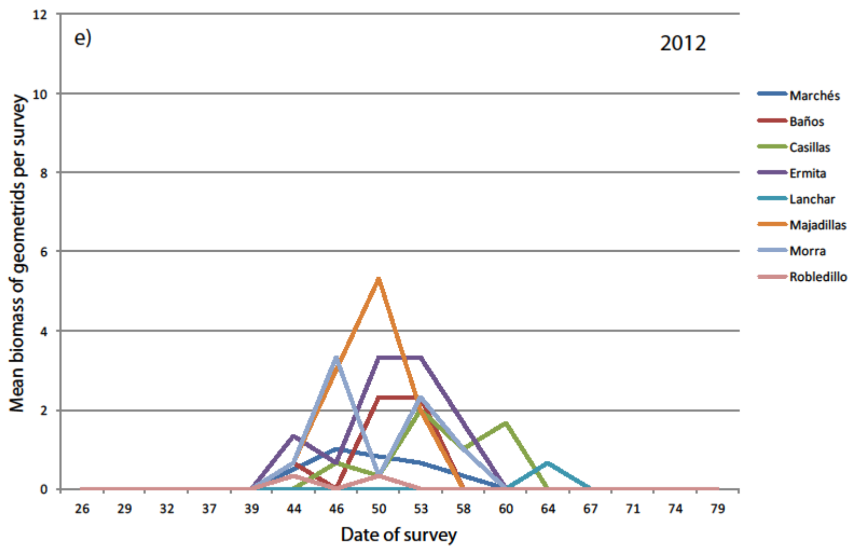
e)
2012
Mean biomass of geometrids per survey
Date of survey
0
2
4
6
8
10
12
26
29
32
37
39
44
46
50
53
58
60
64
67
71
74
79
Marchés
Baños
Casillas
Ermita
Lanchar
Majadillas
Morra
Robledillo

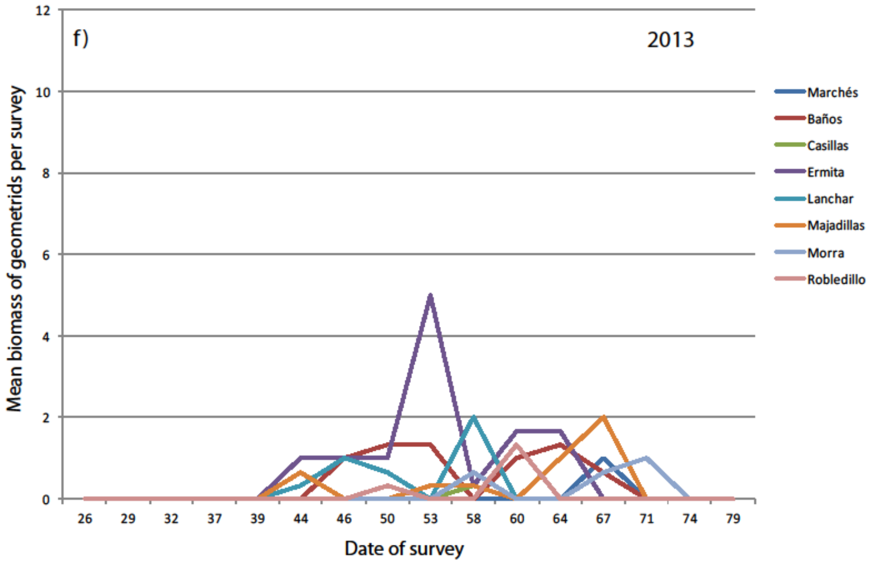
f)
2013
Mean biomass of geometrids per survey
Date of survey
0
2
4
6
8
10
12
26
29
32
37
39
44
46
50
53
58
60
64
67
71
74
79
Marchés
Baños
Casillas
Ermita
Lanchar
Majadillas
Morra
Robledillo

Fig. S2.- Mean nestling weight (±SE) per species (left, blue tit; right, great tit) and year. Sample sizes are shown above the bars.

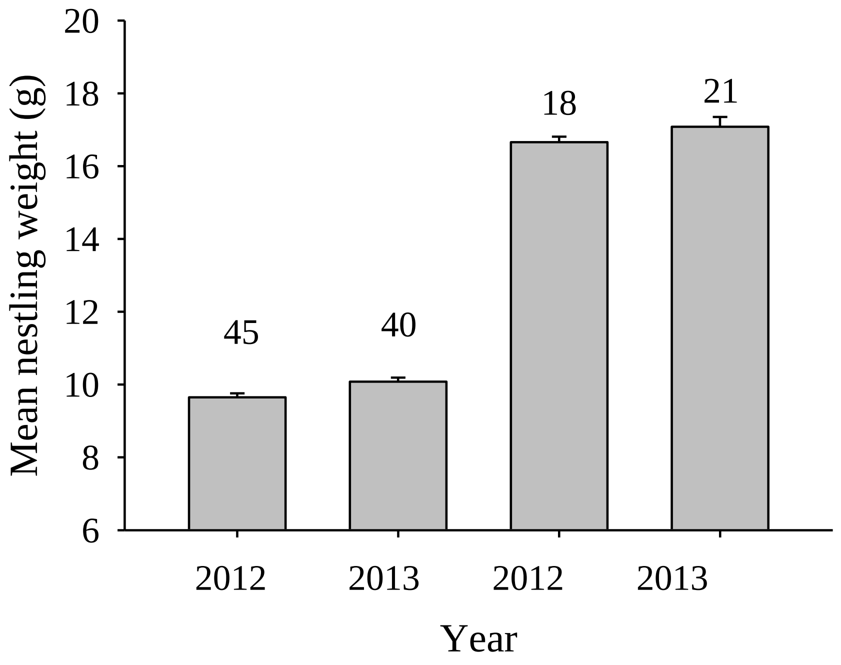

Fig. S3.- Mean fledging rate (±SE) per year. Sample sizes are shown above the bars.

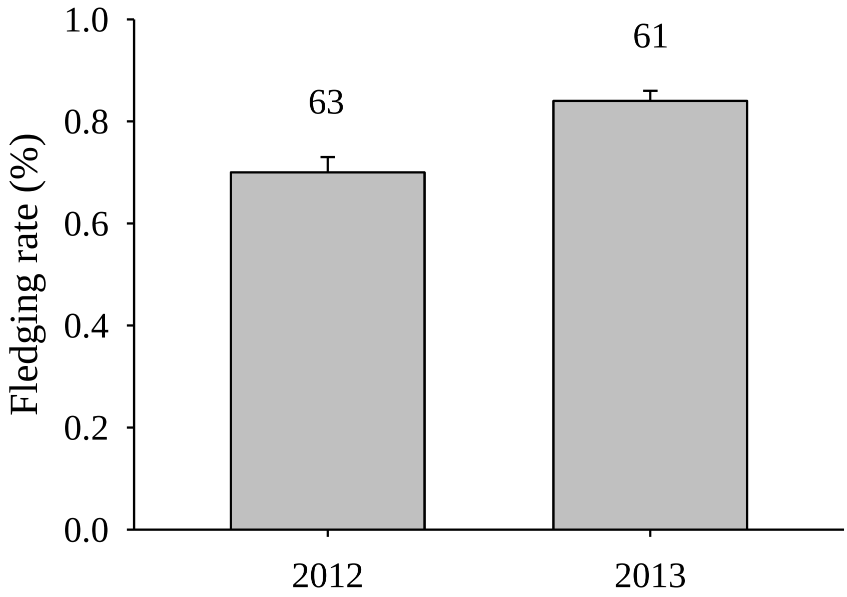

Supplement: Supplementary Material [file srep37750-s1.pdf]
